# Mesorrhine Type of Nose among Medical Students of a Medical College

**DOI:** 10.31729/jnma.8277

**Published:** 2023-09-30

**Authors:** Mina Jha, Ashwini Kumar Jha, Umeshwar Prasad Thakur, Ragni Sinha, Anushree Jha

**Affiliations:** 1 Department of Anatomy, Janaki Medical College and Teaching Hospital, Janakpurdham, Dhanusha, Nepal; 2 Department of Surgery, Janaki Medical College and Teaching Hospital, Janakpurdham, Dhanusha, Nepal; 3 Department of Anatomy, Karnali Academy of Health Sciences, Chandannath, Jumla, Nepal; 4 Department of Pharmacology, Janaki Medical College and Teaching Hospital, Janakpurdham, Dhanusha, Nepal; 5 Kathmandu Cancer Center, Changunarayan, Bhciktapur, Nepal

**Keywords:** *medical students*, *nose*, *prevalence*, *sexual dimorphism*

## Abstract

**Introduction:**

The nose is an attraction point in our face and one of the important sense organs of our body. The knowledge of the type of nose is essential for surgeons undertaking esthetic repair and reconstruction of noses The aim of this study was to find out the prevalence of the mesorhine type of nose among medical students of a medical college.

**Methods:**

A descriptive cross-sectional study was done among medical students of medical college after obtaining ethical approval from the Institutional Review Committee. Students of age groups 16-25 years from different religions with various castes/ethnicities were included. Those with craniofacial abnormalities, nasal deviation and a history of nasal trauma were excluded. All the nasal parameters were measured with the help of vernier's calliper and the nasal index was calculated. Convenience sampling method was used. The point estimate was calculated at a 95% Confidence Interval.

**Results:**

Among 215 medical students, the prevalence of mesorrhine type of nose was 130 (60.46%) (53.92-67, 95% Confidence Interval).

**Conclusions:**

The prevalence of mesorrhine type of nose among medical students was similar to other studies done in similar settings.

## INTRODUCTION

Human physical variability especially facial contour, has always been an interesting subject for anatomists. A pyramidal structure on the centre of the face, is the nose.^1^ It has the external nose and nasal cavity. The external nose is made up of bones and cartilage which maintains its shape and serves a cosmetic purpose.^2^

Various methods are used to study the nose such as morphometric analysis, cephalometry, photography, 3D scans and digitizers.^3^ Nasal index (NI), the ratio of nasal width to nasal height multiplied by 100 is useful in sex determination, distinguishing racial and ethnic differences, nasal analysis, and rhinoplasty.^4^ Anthropometric measurements are useful in comparing different ethnicities.^5^

The aim of this study was to find out the prevalence of mesorrhine type of nose among medical students of a medical college.

## METHODS

This descriptive cross-sectional study was conducted among medical and nursing students studying at Janaki Medical College and Teaching Hospital, Janakpurdham, Dhanusha, Nepal. The study was conducted from 1 Dec 2022 to 30 May 2023. Ethical approval was taken from the Institutional Review Committee of the same institution (IRC/16/2079-80). Informed written and verbal consent was obtained from each participant after explaining the procedure of taking nasal measurements. All medical and nursing students who gave consent for the study were included. Those with craniofacial abnormality, maxillofacial or reconstructive surgery, nasal deviation and a history of nasal trauma were excluded. Convenience sampling method was used. The sample size was calculated by using the following formula:


$$\begin{array}{ll} \text{n} & ={\text{Z}}^{2}\times \frac{\text{p}\times \text{q}}{{\text{e}}^{\text{2}}}\\ & =1.96^{2}\times \frac{0.63\times 0.37}{0.07^{2}}\\ & =183 \end{array}$$

Where,

n = minimum required sample size
Z = 1.96 at 95% Confidence Interval (CI)
p = prevalence taken from the previous study as, 63.04%^1^
q = 1-p
e = margin of error, 7%

The minimum required sample size was 183. However, the final sample size taken was 215.

While taking measurements students were sat in an upright and relaxed mood with the head in an anatomical position to maintain the Frankfurt horizontal line (line joining the infraorbital margin to the external acoustic meatus). Before taking anthropometric measurements, surface landmarks were noted on the face. By using standard anthropometric methods with a digital sliding Vernier's calliper at a precision level of 0.1 mm,^6^ anthropometric data was obtained. Nasal height was measured by placing the upper fixed divider arm of the Vernier's calliper on the nasion of the nose superiorly and then the lower moveable divider arm on the subnasale. The readings were recorded from the digital screen of the calliper. The nasal breadth was measured at a right angle to the nasal height from ala to ala. The measuring techniques followed internationally accepted standards in anthropometry and were taken to the nearest 0.01 cm. The nasal index was calculated as the ratio of nasal width to nasal height multiplied by 100. It was ensured that the calliper was placed properly and accurate readings were taken. It was also ensured that each subject did not smile or change facial expressions while taking measurements in order to get accurate values.^6^

Data obtained was entered into Microsoft Excel 2010 and analyzed using IBM SPSS Statistics Version 20.0. The point estimate was calculated at a 95% CI.

## RESULTS

Among 215 medical students, the prevalence of mesorrhine type of nose was 130 (60.46%) (53.92-67, 95% CI). Among them, 51 (39.23%) were male and 79 (60.77%) were females, with a mean age of 23±0.70 years (minimum: 16, and maximum: 25 years). A total of 119 (91.54%) were Hindu, 6(4.61%) Buddhist and 5 (3.85%) were Muslim (Table 1).

**Table 1:** Sociodemographic characteristics of the students with mesorrhine type of nose (n = 130).

Characteristics	Category	n (%)
Sex	Male	51 (39.23)
	Female	79 (60.77)
Religion	Hindu	119 (91.54)
	Buddhist	6 (4.61)
	Muslim	5 (3.85)
Ethnicity/Caste	Brahmin/Chettri	42 (32.31)
	Madheshi	40 (30.76)
	Janajati/Newar	38 (29.23)
	Dalit	5 (3.85)
	Muslim	5 (3.85)
Stream	MBBS	108 (83.08)
	Nursing	22 (16.92)
Place of Residence	Rural	80 (61.54)
	Urban	50 (38.46)
Country	Nepal	118 (90.77)
	India	12 (9.23)
Ecological residence	Terai	82 (63.08)
	Hill	44 (33.84)
	Mountain	4 (3.08)

The mean nasal index, nasal width and nasal height of students with mesorrhine type of nose was 76.72±10.26, 35.83±2.80 and 46.73±3.02 mm respectively (Table 2).

**Table 2:** Nasal parameters of students with mesorrhine type of nose (n = 130).

Parameters	Mean±SD
Nasal height (mm)	46.73±3.02
Nasal width (mm)	35.83±2.80
Nasal index	76.72±10.26
Nasal length (mm)	41.83±2.60
Nasal depth (mm)	20.15±0.28

## DISCUSSION

The prevalence of mesorrhine type of nose among medical students was 60.46% in the present study. A similar study conducted on medical students in central Nepal showed 63.03% of mesorrhine types of noses.^1^ Another study conducted in the Hindu community of India showed 63.73% and 56.4% students with morphine-type nose studies performed in medical students.^7,8^ Another study of two different ethnic groups Tharu and Mongoloid populations also found the mesorrhine type of nose but the broader nose.^9^ The possibility might be that the genetic makeup of individuals affects the type of nose. In contrast to the study conducted on dental students of Nepal, the prevalence of mesorrhine type of nose was 70.6% which is higher than the present study.^10^ Another study conducted in southern Iran revealed 44.5% in Sistani and 1.5% in Baluch which showed very little value of mesorrhine type of nose.^5^

Nose vary considerably in its shape and size.^11^ In the anthropological field, esthetic surgery, and research work, the anthropometric study of nasal index is regarded as a valuable asset for researchers.^10^ The mean nasal height, width, mean nasal length and depth were 46.73±3.02 mm, 35.83±2.80 mm, 41.83±2.60 mm and 20.15±0.28 mm respectively. From these nasal heights and width, the nasal index was calculated with a mean nasal value of 76.72±10.26. A similar type of study conducted among dental students showed a mean nasal index of 81.34±14.88 whose value was higher than the present study but revealed a similar finding with the present study mesorrhine type of nose.^12^

Various studies have indicated racial and ethnic differences in nasal index amongst different populations.^13^ Many literatures related to nasal morphometry showed variations in nasal parameters between men and women.^14^ Nose shape can give information about race ethnicity, age and sex.^3^ Nasal index is related to regional and climatic differences.^15^ In the present study, students having mesorrhine type of nose were from all three ecological diversity terai, hill and mountain.

However, the study had some limitations as it was conducted in a single centre with a limited number of medical students. So, it cannot be generalized among all the students studying nursing and medical sciences all over Nepal. Further, large-scale studies are needed to be carried out in future to find out the exact prevalence of nose type. There are no such studies conducted in Nepal so far showing the differences in three ecological diversities, further studies are recommended.

## CONCLUSIONS

The prevalence of mesorrhine type of nose among medical students was similar to other studies done in similar settings. Future studies are recommended in the fields of forensic science, reconstructive cosmetic surgery and anthropology.
